# The stress response against denatured proteins in the deletion of cytosolic chaperones *SSA1/2 *is different from heat-shock response in *Saccharomyces cerevisiae*

**DOI:** 10.1186/1471-2164-6-141

**Published:** 2005-10-07

**Authors:** Rena Matsumoto, Kuniko Akama, Randeep Rakwal, Hitoshi Iwahashi

**Affiliations:** 1 Graduate School of Science and Technology, Chiba University, 1-33 Yayoi-cho, Inage, Chiba, Chiba 263-8522, Japan; 2 International Patent Organism Depositary (IPOD), National Institute of Advanced Industrial Science and Technology (AIST), Central 6, 1-1-1 Higashi, Tsukuba, Ibaraki 305-8566, Japan; 3 Human Stress Signal Research Center (HSSRC), AIST, Central 6, 1-1-1 Higashi, Tsukuba, Ibaraki 305-8566, Japan

## Abstract

**Background:**

A yeast strain lacking the two genes *SSA1 *and *SSA2*, which encode cytosolic molecular chaperones, acquires thermotolerance as well as the mild heat-shocked wild-type yeast strain. We investigated the genomic response at the level of mRNA expression to the deletion of *SSA1/2 *in comparison with the mild heat-shocked wild-type using cDNA microarray.

**Results:**

Yeast cDNA microarray analysis revealed that genes involved in the stress response, including molecular chaperones, were up-regulated in a similar manner in both the *ssa1/2 *deletion mutant and the mild heat-shocked wild-type. Genes involved in protein synthesis were up-regulated in the *ssa1/2 *deletion mutant, but were markedly suppressed in the mild heat-shocked wild-type. The genes involved in ubiquitin-proteasome protein degradation were also up-regulated in the *ssa1/2 *deletion mutant, whereas the unfolded protein response (UPR) genes were highly expressed in the mild heat-shocked wild-type. RT-PCR confirmed that the genes regulating protein synthesis and cytosolic protein degradation were up-regulated in the *ssa1/2 *deletion mutant. At the translational level, more ubiquitinated proteins and proteasomes were detected in the *ssa1/2 *deletion mutant, than in the wild-type, confirming that ubiquitin-proteasome protein degradation was up-regulated by the deletion of *SSA1/2*.

**Conclusion:**

These results suggest that the mechanism for rescue of denatured proteins in the *ssa1/2 *deletion mutant is different from that in the mild heat-shocked wild-type: Activated protein synthesis in the *ssa1/2 *deletion mutant supplies a deficiency of proteins by their degradation, whereas mild heat-shock induces UPR.

## Background

Exposure to certain kinds of environmental stress factors, such as chemical, heat, osmotic, etc., induces living organisms to express stress proteins, thereby enabling the organism to acquire stress tolerance. This phenomenon is called the "stress response". Especially, the heat-inducible proteins termed "heat-shock proteins (Hsps)" constitute an important part of the stress-responsive proteins [1]. *HSP70s *(70 kDa HSPs) were discovered in *Drosophila melanogaster*, and their homologs have been found in various organisms including yeast [2,3]. *HSP70s *also function as molecular chaperones [2,3]. In the *Saccharomyces cerevisiae *genome, there are ca. 14 *HSP70*-like genes. The *SSA*, *SSB *and *SSE *families are cytosolic *HSP70 *[4-6], whereas the *SSC1 *is localized to the mitochondria [7,8]. In addition, *KAR2 *(BiP) is localized to the endoplasmic reticulum [9-12]. The *SSA *family contains 4 genes, *SSA1*, *SSA2*, *SSA3 *and *SSA4 *[13]. Not only are the *SSA1 *and *SSA2 *genes constitutively expressed, they are also 96% identical at the nucleotide level [2]. Moreover, there is no change in the phenotype of deletion in either of the *SSA1 *and *SSA2 *genes compared with the wild-type. In addition, they do not show thermotolerance without pre-heat treatment at 37°C [14]. However, the *ssa1/2 *double deletion mutant acquires thermotolerance even at 23°C, and shows a slow growth rate [14]. A suppressor, *EXA3-1 *which is an allele of *HSF1 *encoding a heat shock factor [15,16] recovers its growth rate. This phenomenon in the *ssa1/2 *deletion mutant is speculated to result from the overexpression of certain Hsps [17]. *HSP104 *and *SSA4 *are found to be highly expressed in the *ssa1/2 *deletion mutant [4,18].

*SSA1 *is involved in protein transport and the rescue of denatured proteins [19-22], and possesses ATPase activity [23]. Sti1p activates ATPase activity of Ssa1p [24]. In addition, Hsp70 is a co-chaperone with Hsp104 and Hsp40 in both *S. cerevisiae *and *E.coli *[25,26]. The relationship between these chaperones and human misfolding disease has been shown [27,28]. On the other hand, *SSA2 *is involved in protein transport into the vacuole [29,30]. Thus, *SSA1 *is multi-functional, and the *ssa1/2 *double deletion mutant shows drastic changes needed to acquire thermotolerance, which is similar to the mild heat-shocked wild-type. As Ssa1p and Ssa2p are cytosolic molecular chaperones, it is hypothesized that unfolded proteins appear by the double deletion of *SSA1/2*.

However, genome-wide expression analysis of the *ssa1/2 *deletion mutant using cDNA microarray has not been carried out. We believe that gene expression profiling of the *ssa1/2 *deletion mutant is necessary not only to describe the genomic response developed by yeast to the deletions, but also to reveal the mechanism of the response to denatured proteins. To support the cDNA microarray data, we also performed RT-PCR, and immunoblot analysis of several yeast proteins separated by two-dimensional gel electrophoresis (2-DGE). We demonstrate that the deletion of *SSA1/2 *genes induces up-regulation of the genes involved in both protein degradation and synthesis, whereas mild heat shock induces UPR.

## Results

### Comparison of the mRNA expression profiles between the ssa1/2 deletion mutant and the mild heat-shocked wild-type

**Figure 1:**
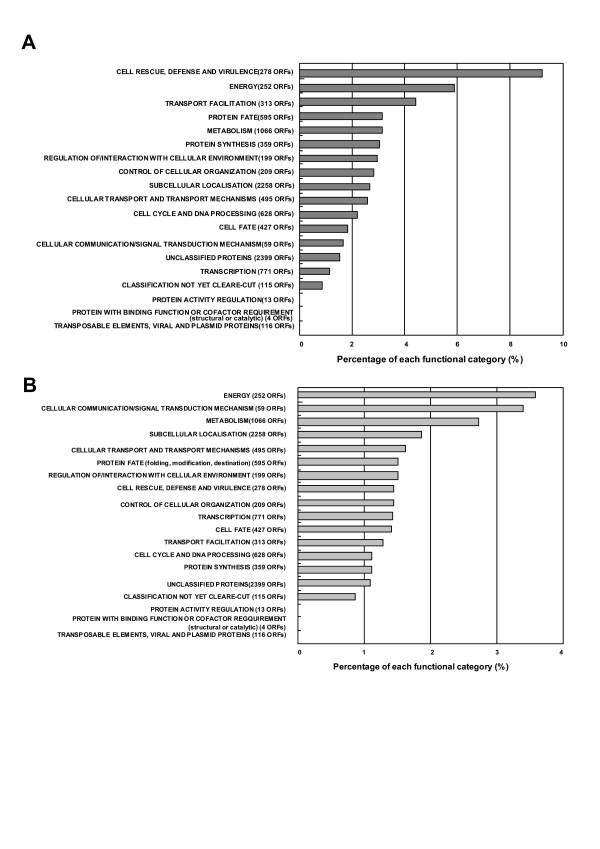
**The overview of expressed genes in the *ssa1/2 *deletion mutant. ***S. cerevisiae *JN14 (*ssa1/2*) and JN54 (wild-type) cells were incubated at 30°C to a logarithmic phase (OD_660 _= 1). The up-regulated genes (over 2- fold expressed) and down-regulated genes (over 2-fold suppressed) in the *ssa1/2 *deletion mutant were determined by twice induction of three individual experiments. These genes were functionally categorized using Comprehensive Yeast Genome Database (CYGD) in Munich International Center of Protein Sequence (MIPS) [52]. A, up-regulated genes; B, down-regulated genes.

**Figure 2:**
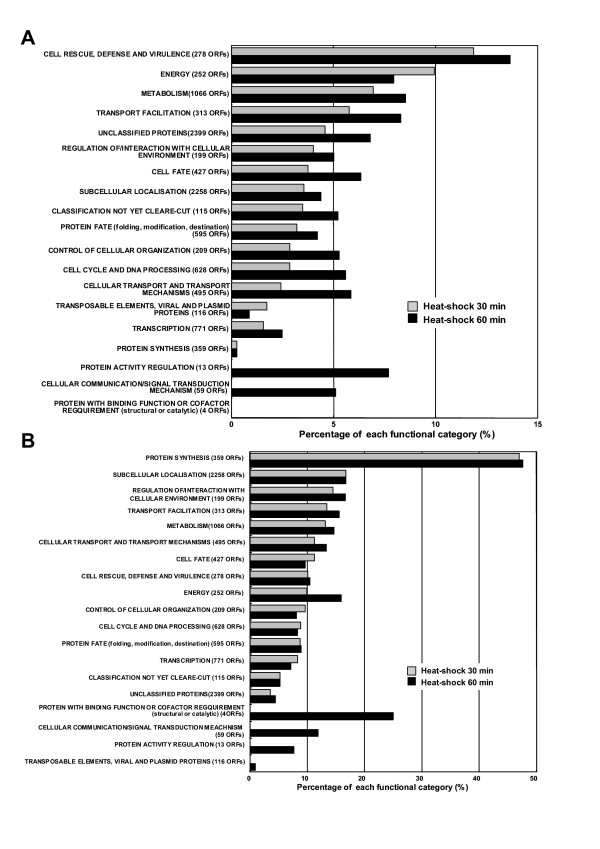
**The overview of expressed genes in the mild heat-shocked wild-type. ***S. cerevisiae *JN54 (wild-type) cells were incubated at 30°C to a logarithmic phase (OD_660 _= 1), and were then treated with mild heat-shock at 43°C for 30 or 60 min. The up-regulated genes (over 2- fold expressed) and down-regulated genes (over 2-fold suppressed) in mild heat-shocked wild-type were determined by twice induction of three individual experiments. These genes were functionally categorized as in Figure 1. A, up-regulated genes. B, down-regulated genes.

To investigate the mechanism of the response to denatured proteins comprehensively, the mRNA expression profiling of the *ssa1/2 *deletion mutant was carried out using yeast cDNA microarray, in comparison with the mild heat-shocked wild-type. The number of up-regulated genes in the *ssa1*/*2 *deletion mutant was 144, while that in the mild heat-shocked wild-type by exposure for 30 and 60 min at 43°C was 274 and 400, respectively. The functionally categorized up-regulated genes are shown in Figs. 1A and 2A. The most highly up-regulated genes were categorized into "Cell rescue, defense, and virulence (stress-inducible proteins)" in both *ssa1*/*2 *and mild heat-shocked wild-type, of which the rates were 8% and 10%, respectively. On the other hand, the number of down-regulated genes in *ssa1/2 *was 94, while that in the mild heat-shocked wild-type by exposure for 30 and 60 min at 43°C was 610 and 643, respectively. The functionally categorized down-regulated genes are shown in Figs. 1B and 2B.

**Figure 3:**
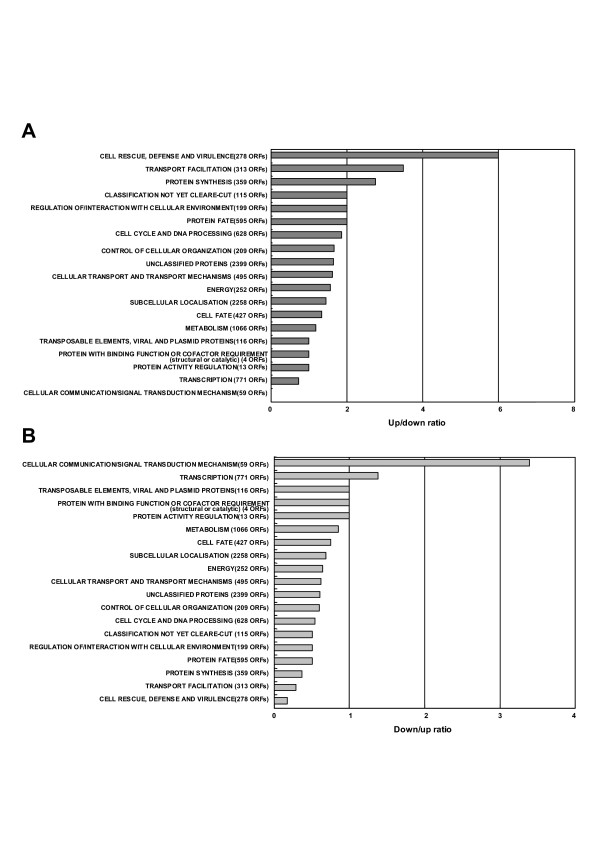
**The comparison between up-regulated genes and down-regulated genes in the *ssa1/2 *deletion mutant. **Functional categories were the same as in Figure 1. The ratios of up/down-regulated genes or down/up-regulated genes were calculated using the percentages of each category in Fig. 1. A, up/down-regulated genes; B, down/up-regulated genes.

**Figure 4:**
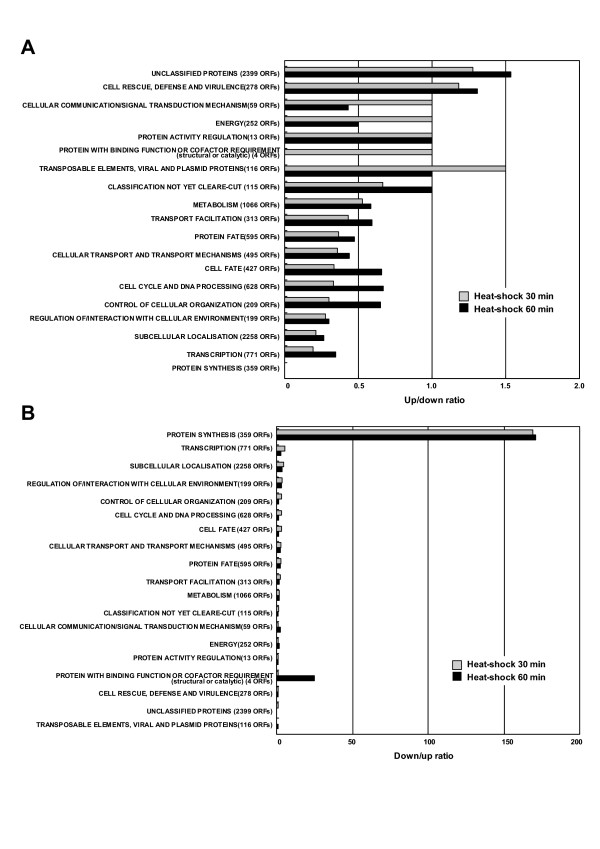
**The comparison between up-regulated genes and down-regulated genes in the mild heat-shocked wild-type. **Functional categories are as given in Figure 1. The ratios of up/down-regulated genes or down/up-regulated genes were calculated using the percentages of each category in Fig. 2. A, up/down-regulated genes; B, down/up-regulated genes.

In the *ssa1*/*2 *deletion mutant, the percentages of up-regulated genes categorized in "Cell rescue defense and virulence", "Transport facilitation" and "Protein fate" was approximately 2–6 times larger than those of the down-regulated genes (Fig. 3A), and the opposite results were found in "Cellular communication/signal transduction mechanism" category (Fig. 3B). In the mild heat-shocked wild-type, there were no categories in which the percentages of up-regulated genes were over 2-times larger than those of the down-regulated genes (Fig. 4A). However, the percentage of down-regulated genes in "Protein synthesis" was particularly larger (ca. 170-times) than that of the up-regulated genes (Fig. 4B). Thus, the number of up-regulated genes in "Protein synthesis" was remarkably smaller than that of the down-regulated genes in the heat-shocked wild type. Conversely, in the *ssa1/2 *deletion mutant, the number of up-regulated genes in "Protein synthesis" was larger than that of the down-regulated genes. Therefore, we focused on protein synthesis and correlated protein fate as well as "Cell rescue, defense and virulence" in the *ssa1/2 *deletion mutant.

**Figure 5:**
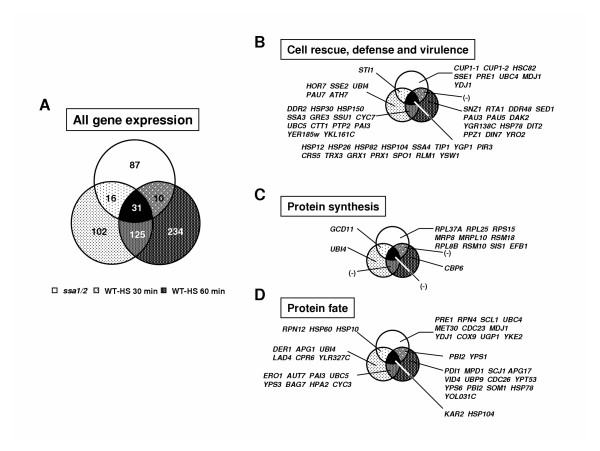
**The comparison of up-regulated genes in the *ssa1/2 *deletion mutant with those in mild heat-shocked wild-type. **Venn Diagrams were constructed by the "GeneSpring" software (Silicon Genetics). Functional subcategories are according to Figure 1.

Figure 5 shows a detailed comparison of these categorized genes up-regulated in the *ssa1/2 *deletion mutant and in the mild heat-shocked wild-type. Figure 5A shows the comparison of all the genes up-regulated in the *ssa1/2 *deletion mutant and the mild heat-shocked wild-type. In the categories of "Cell rescue, defense and virulence", several Hsps, including molecular chaperones, were commonly up-regulated in the *ssa1/2 *deletion mutant and the mild heat-shocked wild-type (Fig. 5B). Although genes related to protein synthesis were greatly suppressed in the mild heat-shocked wild-type, ribosomal protein genes were found to be up-regulated only in the *ssa1/2 *deletion mutant (Fig. 5C). Table 1 shows the expression level of these genes: *RPL37A*, *RPL25*, *MRP8*, *RPS15*, *MRPL10*, *RSM18*, *RPL8B *and *RSM10*.

**Table 1:** The genes involved in ribosomal proteins up-regulated in the *ssa1/2 *deletion mutant.

Gene name	Expression level*	Description
*RPL37A*	3.2	60S ribosomal protein L37A (YL35)
*RPL25*	2.8	Ribosomal protein L25 (rpl6L)
*MRP8*	2.4	Mitochondrial ribosomal protein
*RPS15*	2.2	40S ribosomal protein S15 (S21) (rp52) (RIG protein)
*MRPL10*	2.0	Mitochondrial ribosomal protein MRPL10 (YmL10)
*RSM18*	1.9	Protein of the small subunit of the mitochondrial ribosome
*RPL8B*	1.8	Ribosomal protein L8B (L4B) (YL5)
*RSM10*	1.7	Protein of the small subunit of the mitochondrial ribosome

*The expression levels are the average value of three independent experiments.

On the other hand, in the category of "Protein fate", the *PRE1*, *RPN4*, *RPN12 *and *SCL1 *genes that encode for cytosolic proteasome subunits, were found to be up-regulated in *ssa1/2 *deletion mutant (Fig. 5D). In addition, the *UBC4 *(ubiquitin conjugating enzyme) gene was also up-regulated. Table 2 shows the expression level of the genes involved in protein degradation. These results suggest that the ubiquitin-proteasome protein degradation pathway is activated in the *ssa1/2 *deletion mutant. Although a few ubiquitin-proteasome genes (*UBI4*, *UBC5 *and *UBP9*) were up-regulated in the mild heat-shocked wild-type, they were not in common with those up-regulated in the *ssa1/2 *deletion mutant (Fig. 5D). The proteasome genes up-regulated in the mild heat-shocked wild-type included vacuolar protein genes (*AUT7*, *LAD4 *and *APG17*), and unfolded protein response (UPR) genes (*DER1*, *PDI1 *and *ERO1*) (Fig. 5D).

**Table 2:** The genes involved in proteolytic degradation up-regulated in the *ssa1/2 *deletion mutant.

Gene name	Expression level*	Description
*CDC23*	3.7	Cell division cycle protein
*PRE1*	3.0	22.6 kDa proteasome subunit (20S proteasome subunit C11 (beta4))
*UBC4*	2.8	Ubiquitin-conjugating enzyme
*RPN4*	2.6	Ubiquitin-mediated 26S proteasome subunit
*MET30*	2.5	Met30p contains 5 copies of WD40 motif and interacts with and regulates Met4p
*SCL1*	2.4	20S proteasome subunit YC7alpha/Y8 (protease yscE subunit 7)
*PBI2*	2.3	Proteinase inhibitor that inhibits protease Prb1p (yscB)
*RPN12*	2.0	26S proteasome regulatory subunit

*The expression levels are the average value of three independent experiments.

### Confirmatory RT-PCR for proteolytic degradation- and ribosomal biogenesis-related genes

**Figure 6:**
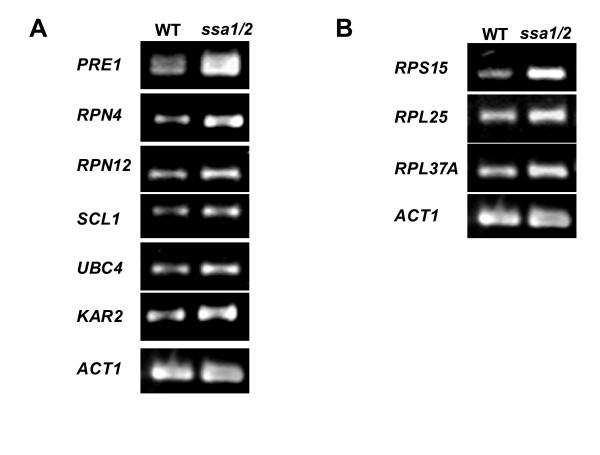
**RT-PCR analysis of ribosomal protein and proteolytic degradation genes in the *ssa1/2 *deletion mutant and wild-type. **RT-PCR was carried out as described in "Materials and Methods", and primers, product size and numbers of PCR cycle are described on Table 3. The RT-PCR products were run on a 4% Nu-Sieve 3:1 Plus agarose gel. A, genes for proteolytic degradation. B, ribosomal protein genes.

To verify that both protein synthesis and degradation are activated in the *ssa1/2 *deletion mutant, RT-PCR analysis of several proteolytic degradation genes and cytosolic ribosomal protein genes was carried out. Proteasome subunit genes (*PRE1, RPN4, RPN12*, and *SCL1*), an ubiquitin conjugating enzyme gene (*UBC4*), and cytosolic ribosomal protein genes were found to be up-regulated in the *ssa1/2 *deletion mutant compared with the wild-type (Fig. 6A and Fig. 6B). This result supports the cDNA microarray data showing that both ubiquitin-proteasome protein degradation and protein synthesis were activated by deletion of the *SSA1*/*2 *genes. Only *KAR2 *was highly expressed among the UPR genes (Fig. 6A).

### Immunoblot analysis of proteolytic degradation-related gene products

**Figure 7:**
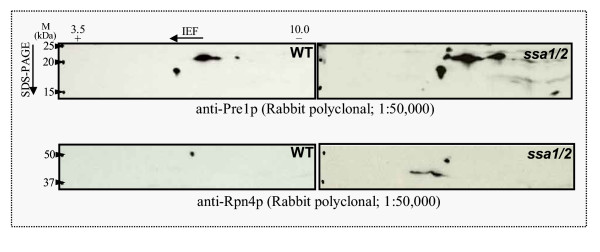
**Immunoblot analysis of proteasome subunit genes in the *ssa1/2 *deletion mutant and wild-type. **2-DGE was performed as described in "Material and Methods", followed by immunoblot analysis. A, Pre1p (20 S proteasome subunit). B, Rpn4p (Ubiquitin-mediated 26 S proteasome subunit).

**Figure 8:**
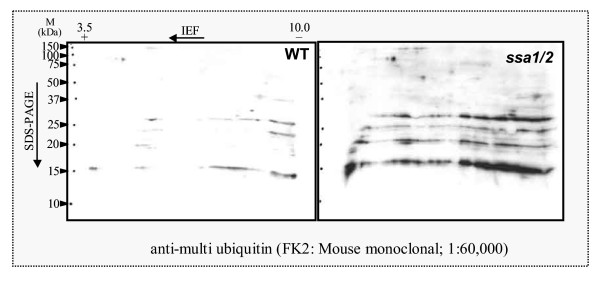
**Ubiquitinated proteins in the *ssa1/2 *deletion mutant and wild-type. **2-DGE was performed followed by immunoblot using an anti-multi ubiquitin mouse monoclonal antibody (FK2).

To confirm that ubiquitin-proteasome protein degradation is activated at the translational level in the *ssa1/2 *deletion mutant, immunoblot analysis was performed. Pre1p (20 S proteasome subunit) and Rpn4p (Ubiquitin-mediated 26 S proteasome subunit) increased in the *ssa1/2 *deletion mutant compared with the wild-type (Fig. 7). An anti-multi ubiquitin mouse monoclonal antibody (FK2) [31,32] detects only ubiquitin that is covalently bound to substrate proteins, i.e. ubiquitinated proteins, and not free ubiquitin [31,32]. Ubiquitinated proteins especially with molecular weights less than 30 kDa increased in the *ssa1/2 *deletion mutant in comparison with the wild-type (Fig. 8).

## Discussion

In the present study, we reveal global differences in gene expression between yeast cells lacking two cytosolic *HSP70*s, *SSA1 *and *SSA2*, and the mild-heat-shocked wild-type using cDNA microarray technologoly.

Results from cDNA microarray analysis reveal that the stress-inducible protein genes, including molecular chaperones, were up-regulated in the *ssa1/2 *deletion mutant in a similar fashion as seen in the mild heat-shocked wild-type (Figs. 1A, 2A, and 5B). It is clear that thermotolerance is due to expression of these stress-inducible proteins. In the *ssa1/2 *deletion mutant, *HSF1 *suppressing growth rate of the *ssa1/2 *[15,16] was expressed normally and its expression level was unchanged (data not shown). Several genes involved in the ubiquitin-proteasome protein degradation pathway were up-regulated in the *ssa1/2 *deletion mutant (Fig. 5D and Table 2). *UBC4 *[33,34] was also up-regulated in the *ssa1/2 *deletion mutant, which is consistent with a previous report [35]. *UBC4/5 *is necessary for binding between the substrates and Lys48 of ubiquitin that is a target of the 26 S proteasome [34], and for binding between the substrates and Lys63 of ubiquitin, that is not a target of the 26 S proteasome. In addition to *UBC4*, we found up-regulation of several proteasome genes (*PRE1*, *RPN4*, *RPN12 *and *SCL1*) in the *ssa1/2 *deletion mutant. *PRE1 *and *SCL1 *encode 20 S proteasome, and *RPN4 *and *RPN12 *encode 26 S proteasome [36]. *RPN4 *(*SON1*) is a factor involved in ERAD (endoplasmic reticulum associated degradation) [37]. All these genes are essential for degradation of the ubiquitinated proteins [36,38]. RT-PCR data support the up-regulation of these proteasome genes by the deletion of *SSA1/2 *(Fig. 6A). Moreover, we confirmed that Pre1p and Rpn4p were up-regulated in the *ssa1/2 *deletion mutant at the translational level by immunoblotting (Fig. 7). This result provided further evidence that proteolytic degradation by proteasomes was stimulated by the deletion of *SSA1/2*. As shown in Fig. 8, more ubiquitinated proteins, especially with molecular weights less than 30 kDa, were detected in the *ssa1/2 *deletion mutant than in the wild-type. The deletion of *UBP3 *in *ssa1/2 *has been reported to lead to a significant increase in the number of the ubiquitinated proteins, mainly with molecular weights of more than 30 kDa [35]. The expression level of *UBP3 *did not change in the *ssa1/2 *deletion mutant compared with the wild-type (data not shown). Therefore, the increase of ubiquitinated proteins in *ssa1/2 *is not caused by the deletion of *UBP3*. There are two possibilities for the increase of ubiquitinated proteins in the *ssa1/2 *deletion mutant. First, insufficiency of the UPR in the *ssa1/2 *deletion mutant may lead to activation of the ubiquitin-proteasome protein degradation system. Thus, ubiquitination of the target proteins increases and the expression of proteasome genes is induced. Second, some defect of deubiquitination occurs in the *ssa1/2 *deletion mutant, consequently leading to the accumulation of ubiquitinated proteins in the cell followed by cell death. Furthermore, proteolytic degradation by proteasomes is facilitated. On the other hand, we found that several genes encoding ribosomal proteins were up-regulated in the *ssa1/2 *deletion mutant (Figs. 5C, 6A, and Table 1), implying that protein synthesis is activated by the deletion of *SSA1/2*.

In case of the mild heat-shocked wild-type, genes involved in protein synthesis were significantly suppressed (Figs. 2B and 4B), and the proteasome genes up-regulated in the *ssa1/2 *deletion mutant did not show any change in their expression levels (Fig. 5D). Instead, some UPR genes (*PDI1*, *DER1*, *ERO1 *and *KAR2*) were up-regulated (Fig. 5D), implying that UPR occurs during mild heat-shock. The mechanism of UPR is known to induce the up-regulation of ER chaperones for refolding when unfolded proteins accumulate in the ER [39]. In the *ssa1/2 *deletion mutant, the expression of three UPR genes (*PDI1*, *DER1 *and *ERO1) *remained unchanged (data not shown), and only *KAR2 *was up-regulated (Fig. 3).

Gasch *et al*. has reported the genome-wide expression analysis of yeast cells exposed to environmental changes [40]. We compared our data on the mild heat shocked wild-type yeast cells with their data on the wild-type cells shifted to 37°C from 25°C. The stress-inducible protein genes up-regulated in the mild-heat shocked wild type (*SSA3, SSA4, SSE2, CTT1, HSP26, HSP78, and HSP104) *(Fig. 5B) are in common with the results obtained by Gasch *et al*. [40]. In the category of "Protein fate", more than 70% of the up-regulated genes in our experiments are also in common with their results [40], even though there is a time lag with their experiments. However, *DER1*, one of the UPR genes, was not found to be up-regulated in their study during the entire the heat-shock treatment period [40]. In contrast, *DER1 *was up-regulated in our experiments (Fig. 5D). This may be due to the difference in the temperature and time of heat-shock treatment. On the other hand, in the category of "Protein synthesis", ribosomal protein genes are significantly suppressed in their experiments [40], which is consistent with our data. From these comparisons, it can be said that our data on the mild heat-shocked wild-type is similar to that reported by Gasch *et al*. [40] in the categories of "Cell rescue, defense, and virulence", "Protein fate" and "Protein synthesis".

It is reasonable that UPR is activated and protein synthesis is suppressed in the mild heat-shocked wild-type. We speculate on the reasons as to why the genes involved in both protein degradation and protein synthesis are up-regulated in *ssa1*/*2 *deletion mutant. In the normal state, proteins are synthesized on the ribosome, followed by post-translational modifications in the ER or the Golgi apparatus to finally become mature and functional entitles. Schubert et al [41] showed that 30% of the de novo synthesized proteins are degraded before coming to maturity. Therefore, it can be reasoned that post-translational protein denaturation occurs moderately even under normal conditions. However, organisms have developed several mechanisms in their response to the denatured proteins. UPR is one of the ER quality control mechanisms [39]. In addition, the refolding of denatured proteins is carried out by cytosolic chaperones [25,42,43], including *SSA1/2 *[20,21]. It can be hypothesized that the deletion of *SSA1/2 *leads to the suppression of refolding, which is then followed by an accumulation of the denatured proteins in cells. The genes involved in proteolytic degradation may be up-regulated to remove such denatured proteins. However, if the ubiquitin-proteasome system keeps on degrading proteins, the depletion of the proteins essential for growth and development will occur. It is suggested that protein synthesis is activated to supply the proteins deleted by proteolytic degradation in the *ssa1/2 *deletion mutant. In the *ssa1/2 *deletion mutant, several hexose transporter genes (*HXT2*, *HXT4*, *HXT6*, *HXT7*), and the genes that belong to early part of glycolysis (*GLK1*, *HXK1*) were up-regulated (data not shown). The expression of these genes, involved in energy generation, may be required for sustaining the increased protein synthesis in the *ssa1/2 *deletion mutant. *HXT *genes up-regulated in the *ssa1/2 *deletion mutant are low-glucose dependent [44-46]. It is possible that the uptake of glucose is activated to generate energy, because energy is consumed by protein synthesis that is induced by the deletion of *SSA1/2*.

These results indicate that different mechanisms of the response to denatured proteins are employed between the *ssa1/2 *deletion mutant and the mild heat-shocked wild-type even though several up-regulated Hsps (molecular chaperones) are common between the *ssa1/2 *deletion mutant and the mild heat-shocked wild-type (Fig. 5B). When Hsp104p, Ydj1p (yeast Hsp40p), and Ssa1p exist together, their chaperone activities increase significantly [25]. From this, it is suggested that the deletion of *SSA1/2 *induces the suppression of their chaperone activities. Recently, the cooperation of Hsp26p wih Hsp104p/Hsp70p/Hsp40p chaperone system on protein disaggregation in yeast was reported [47,48]. Hsp26p co-aggregated with substrate is suggested to be a target of the Hsp104p/Hsp70p/Hsp40p chaperone system [47,48]. Although Ssa1p is able to disaggregate the early Hsp26p-substrate complex (small soluble aggregates), Hsp104p is essential in refolding the late Hsp26p-substrate complex (big insoluble aggregates) [47,48]. Moreover, excess or stoichiometric Hsp26p against denatured substrates is essential for effective refolding [47]. In the *ssa1/2 *deletion mutant, an increase in the mRNA expression levels of *HSP104 *and *HSP26 *was seen (Fig. 5B). Although the refolding of denatured proteins is sure to succeed if *HSP104*/Hsp104p and *HSP26*/Hsp26p are highly expressed, it is a fact that the ubiquitin-proteasome degradation system is facilitated in the *ssa1/2 *deletion mutant. It can be speculated that as constitutive protein denaturation occurs, the ubiquitin-proteasome degradation system is required in addition to the chaperone refolding system in the *ssa1/2 *deletion mutant. Furthermore, there is a possibility that protein refolding by molecular chaperones and ubiquitin-proteasome protein degradation are related. In mammalian cells, the following model has been reported; denatured proteins are refolded by Hsp70-HSP40 chaperone-mediated maturation pathway under the treatment of Hsp90 inhibitor, and then denatured proteins are degraded by ubiquitin-proteasome [49]. It is interesting to note that the ubiquitin-proteasome protein degradation system in yeast is induced when the chaperone function is inhibited by the deletion of *SSA1/2*. However, at present, our data are not sufficient to propose a similar model in yeast, and this remains a topic for future study.

## Conclusion

The protein synthesis and ubiquitin-proteasome degradation system were up-regulated in the *ssa1/2 *deletion mutant, whereas UPR genes were up-regulated but protein synthesis was strongly suppressed in the mild heat-shocked wild-type. These results suggest that the mechanism for rescue of denatured proteins in the *ssa1/2 *deletion mutant differs from that in the mild heat-shocked wild-type, although the phenomena on acquisition of thermotolerance are similar.

## Methods

### Strains and growth condition

*S. cerevisiae *JN14 is the *ssa1/2 *deletion mutant strain (*MATa his3-11, 3-15 leu2-3, 2-112 ura3-52 trp1-?1 lys2? Ssa1-3::HIS3 ssa2-2::URA3*) [15]. *S. cerevisiae *JN54 (*MATa his3-11, 3-15 leu2-3, 2-112 ura3-52 trp1-?1 lys2?*) is the parent strain (wild-type) of the *ssa1/2 *double mutant [15]. Yeast cells were incubated in 100 ml of YPD (1% yeast extract, 2% polypeptone and 2% glucose) medium at 30°C to a logarithmic phase (OD_660 _= 1) using 500 ml Erlenmeyer flasks, and collected by centrifugation (2,800 × g). Cells were washed with distilled water three times, and stocked in a -80°C deep freezer until used for total RNA extraction.

### Heat-shock treatment

*S. cerevisiae *JN54 (wild-type) cells were incubated in YPD medium at 30°C to a logarithmic phase (OD_660 _= 1), followed by treatment with mild heat-shock at 43°C for 30 or 60 min in pre-warmed (43°C) 100 ml of YPD medium using 500 ml Erlenmeyer flasks. Heat-shocked cells were collected by centrifugation. Cells were washed with distilled water three times, and stocked in a -80°C deep freezer until used for total RNA extraction.

### RNA extraction and hybridization to a cDNA microarray

Total RNA was extracted by the hot-phenol method [50]. The extraction of mRNA and reverse-transcription to cDNA was done according to Momose and Iwahashi [51]. Poly (A) +RNA was purified from total RNA with an Oligotex-dT30 mRNA purification kit (TaKaRa, Otsu, Shiga, Japan). Fluorescence-labeled cDNA was synthesized with a Cyscribe cDNA Labeling Kit (Amersham Biosciences, Little Chalfont, Buckinghamshire, UK) and 0.5 mM Cy3-UTP (Amersham Biosciences) or 0.5 mM Cy5-UTP. Cy3-UTP was used for the wild-type (as control), and Cy5-UTP was used for the *ssa1/2 *deletion mutant (as sample). For the heat-shock experiment, Cy3-UTP was used for control cells (30°C), and Cy5-UTP was used for mild heat-shocked cells (43°C). Synthesized cDNA were hybridized to a Kuhara DNA chip (DNA Chip Research, Yokohama, Kanagawa, Japan) at 65°C for 48 h.

### cDNA microarray analysis

Hybridized cDNA microarray were washed, dried, and scanned using Scanarray 4000 (GSI Lumonics, Billerica, MA, USA). Quantification of gene expression was done using the Genepix ver. 4.0 quantitative microarray analysis application program (Axon Instruments, Union City, CA, USA). The ratio of intensity Cy5/Cy3 was calculated and normalized with negative control spots. All the calculations and normalizations were done using "Chip Cleanser" program [52]. The functional categorization of genes was performed using GeneSpring (Silicon Genetics, Redwood City, CA, USA), and Comprehensive Yeast Genome Database (CYGD) at the Munich International Center of Protein Sequence (MIPS) database [52]. The over 2- fold expressed genes by the deletion of *SSA1/2 *or the mild heat-shocked treatment in the wild-type were selected as up-regulated genes and determined by at least twice- induced, out of three individual experiments. Similarly, the over 2- fold suppressed genes by the deletion of *SSA1/2 *or the mild heat-shocked treatment in the wild-type were selected as down-regulated genes, and determined by twice suppressed, out of three individual experiments [51,53]. The values for up- or down-regulated genes were the average ratio from three independent experiments. The data obtained in this experiment are available with the accession numbers GSE3315 (*ssa1/2 *deletion mutant) and GSE3316 (mild heat-shocked wild-type) in the Gene Expression Omnibus Database (GEO) [54].

### RT-PCR analysis

Total RNA extraction was carried out as described above. RT-PCR was performed using the One Step RNA PCR Kit (AMV) (TaKaRa), according to the instructions provided by the manufacturer. The primers used for RT-PCR are described in Table 3, and 0.1 μg of total RNA were used for RT-PCR. After reverse transcription, samples were subjected to a cycling regime of 20–25 cycles (details are mentioned in Table 3). Five μl of RT-PCR products were loaded into the wells of a 4% Nu-Sieve 3:1 Plus agarose (Cambrex Bio Science Rockland, Inc. Rockland, ME, USA) gel, and electrophoresis was carried out for 50 min at 100 V. The gels were stained using 10 μg/ml ethidium bromide followed by visualization of the stained bands with an UV-transilluminator (ATTO, Tokyo, Japan).

**Table 3:** List of primers for RT-PCR

Gene name	Forward primer (5'-3')	Reverse primer (5'-3')	Product size (bp)	No. of cycles
*PRE1*	TGACTTCCAGGCACAGTGAA	TCTCACTCTGCCAACAAAAA	187	25
*RPN4*	CGAAGCATGAAGATTTGTCG	AAGAACATTCCTGAATGCAGAT	202	25
*RPN12*	CCAATCAAAGGAGAAAGCTGA	CTCCGGGAGAGAAAAAGTTG	178	22
*SCL1*	AGTCGGTGTCGCTACAAAGG	CGACAAAAGGGCTTGAAAAG	229	20
*UBC4*	CAGCCAGAGAATGGACAAAGA	AGGTTCCCCTGTACTGTTGC	220	20
*KAR2*	GTTCTGGTGCCGCTGATTAT	CGAAAATTGTATGAAGCTCGAA	205	20
*RPS15*	AGAGCCGGTGCTACTACTTCC	CGTGTACAACCCCCATTCAC	200	22
*RPL25*	CGTTACCAAGAAGGCTTACG	CGTGCACTCTGCCACTACAC	203	22
*RPL37A*	CAAACCGGCTCTGCTTCTAA	TTCCCGTAAGCACTCAAAGG	194	25
*ACT1*	CCTTCCAACAAATGTGGATCT	CAGTGCTTAAACACGTCTTTTCC	200	25

### Antibodies

Anti-Pre1p peptide (Res. No., 17-38) and anti-Rpn4p peptide (Res. No., 499-509) rabbit polyclonal antibodies were ordered to Sigma Genosys (Tokyo, Japan). The anti-multi ubiquitin mouse monoclonal antibody (FK2, Cat. No. SPA-205) was purchased from Stressgen Bioreagents Ltd. Partnership (Victoria, B.C., Canada) [31,32].

### Two-dimensional gel electrophoresis (2-DGE) and Immunoblot analysis

Yeast cells were washed with distilled water three times. Total protein was extracted from cells homogenized with lysis buffer [(7 M urea (ICN Biomedicals, Aurora, OH, USA), 2 M thiourea (Sigma, St. Louis, MO, USA), 4% CHAPS (Sigma), 1% carrier ampholyte (pH 3.5-10, Amersham Biosciences), 18 mM Tris-HCl, pH 7.5, 14 mM Trizma base (Sigma), EDTA-free Proteinase Inhibitor (Roche Diagnostics, Manheim, Germany), 0.2 % Triton X-100, reduced (Sigma), 14.4 mM DTT (Sigma)]. Resuspended cells were broken with glass beads at 4°C for 10 min, and centrifuged at 20,000 × g for 10 min. Cell lysate was centrifuged again at 20,000 × g for 7 min. Equal amounts (350 μg) of protein were subjected to 2-DGE, following O'Farrell's method [55]. Electrophoresis [IEF, carried out in a glass capillary tube of 13 cm length and 3 mm diameter (Nihon Eido, Tokyo, Japan) and SDS-PAGE (12.5% or 15% polyacrylamide gel, 5% stacking and 12.5% or 15% separation gel; using standard glass gels plates obtained from Nihon Eido) in the second dimension] was carried out at a constant current of 35 mA for 2-1/2 h or until the dye (250 μL BPB; 0.1% (w/v) in 10% (v/v) glycerol in MQ) reached the bottom of the gel [56]. Ten μL of the commercially available "ready-to-use" molecular mass standards (Precision Plus Protein Standards, Dual Color, Bio-Rad, Hercules, CA, USA) were loaded next to the acidic end of the IEF tube gel. Reproducibility of 2-DGE protein profiles was confirmed by running at least 3 independent protein samples extracted from the cells of wild-type and the *ssa1/2 *deletion mutant. Electrotransfer of proteins on gel to a PVDF (NT-31, Nihon Eido) membrane was carried out at 1 mA/cm^2 ^with a semi-dry blotter (Nihon Eido) as described previously [57], followed by immunostaining using antibodies (described above). The anti-Pre1p and anti-Rpn4p rabbit polyclonal antibodies were diluted to 1:50,000, and anti-multi ubiquitin mouse monoclonal antibody (FK2) was diluted to 1:60,000. The ECL plus Western Blotting Detection System protocol for blocking, primary and secondary antibody (anti-Rabbit IgG, Horseradish peroxidase linked whole antibody; from donkey) incubation was followed exactly as described (Amersham Biosciences). Immunoassayed proteins were visualized on an X-ray film (X-OMAT AR, Kodak, Tokyo, Japan) using an enhanced chemiluminescence protocol according to the manufacturer's directions (Amersham Biosciences).

## List of abbreviations

SSA: stress seventy family A

RT-PCR: reverse transcription polymerase chain reaction

HSP: heat-shock protein

2-DGE: two-dimensional gel electrophoresis

CHAPS: 3- [(3-cholamidopropyl) dimethylaminol]-1-propanesulfonate

EDTA: ethylenediaminetetraacetic acid

DTT: dithiothreitol

IEF: isoelectric focusing

SDS-PAGE: sodium dodecyl sulfate-polyacrylamide gel electrophoresis

PVDF: polyvinylidene difluoride

CYGD: Comprehensive Yeast Genome Database

MIPS: Munich International Center of Protein Sequence

GEO: Gene Expression Omnibus Database

UPR: unfolded protein response

ERAD: endoplasmic reticulum associated degradation

## Authors' contributions

RM planned and designed the study, performed the experiments and the data analysis, wrote the main draft of the paper, and generated the figures. KA organized all the research, and provided advice for preparing the manuscript. RR designed the protein experiments and RT-PCR analysis, and contributed in figure making and in editing the manuscript. HI planned all the research and designed the experiments, and suggested the draft of the paper. All authors read and approved the final manuscript.
